# Editorial: Enhancing oral health literacy and quality of life: strategies for a healthier future

**DOI:** 10.3389/froh.2026.1784536

**Published:** 2026-01-26

**Authors:** Dhelfeson Willya Douglas-de-Oliveira, Frederico Santos Lages

**Affiliations:** 1 School of Dentistry, Universidade Federal dos Vales Jequitinhonha e Mucuri, Diamantina, Brazil; 2 Laboratory MEC.IM UFMG, School of Dentistry, Universidade Federal de Minas Gerais, Belo Horizonte, Brazil

**Keywords:** global oral health trends, oral health equity, oral health policy, oral health quality of life, patient-centered dental outcomes

## Introduction

Oral health is a fundamental component of overall health, encompassing biological integrity, functional ability and psychosocial comfort (1). Oral diseases and functional impairments affect essential daily activities (from eating and speaking to engaging in social life) and are closely intertwined with an individual's quality of life (2). These outcomes are not determined solely by clinical status but are shaped by a group of factors including oral health literacy (the capacity to obtain, process, and apply oral health information) and the broader social, economic, and cultural contexts in which individuals live (3).

The Research Topic *Enhancing Oral Health Literacy and Quality of Life: Strategies for a Healthier Future* was established to unify interdisciplinary perspectives on how literacy-centered approaches can inform of equitable, sustainable improvements in oral health outcomes. The nine contributions in this Research Topic deepen the understanding of how oral health literacy interacts with functional oral health, health behavior, and social determinants, advancing both theoretical insight and practical strategies to improve quality of life.

## Bridging function and quality of life

A central theme emerging from the collection is the relevance of oral function to health outcomes among older adults. The reviewed evidence highlights that oral health extends beyond clinical indicators to include functional aspects essential for daily living. This perspective is reflected in the systematic overview of oral frailty among hospitalized older adults, which identifies functional decline, such as impaired mastication and swallowing, as a common and clinically significant condition in this population. The synthesis presented in *Systematic review and meta-analysis of oral frailty prevalence among older hospitalized patients* emphasizes the high prevalence of oral frailty and supports the integration of functional oral assessments into hospital care and research, given their association with adverse health outcomes.

Similarly, the cross-sectional analysis in *Prevalence and influencing factors of oral frailty among middle-aged and older patients with chronic kidney disease* shows how a chronic systemic condition such as chronic kidney disease is associated with a high prevalence of oral frailty and multiple influencing factors, including denture use, dry mouth, educational level, self-care ability, and fatigue symptoms. Rather than describing direct causal pathways linking literacy and care behaviors to quality of life, this study highlights the complex interplay between systemic health status and oral functional decline, suggesting that systemic disease can exacerbate oral frailty and related functional impairments. Together, these studies reinforce that functional outcomes (e.g., chewing and swallowing ability) rather than the mere presence of disease alone, provide valuable insight into everyday oral health challenges and their potential impacts on overall health and well-being.

## Oral health literacy as an enabler of engagement

Understanding and improving oral health literacy requires attention to contextual, cultural, and communicative factors. This is illustrated in *Harnessing technology to enhance oral health literacy among Afghan women: an interprofessional community-engaged initiative*, which describes a culturally tailored educational intervention delivered through accessible technology platforms. The study reports improvements in oral health knowledge and selected self-reported oral hygiene practices among Afghan refugee women, underscoring the potential value of culturally sensitive content, community engagement, and interprofessional collaboration in literacy-oriented initiatives targeting underserved populations.

In parallel, *Knowledge, attitudes, and practices toward halitosis among dental patients at Zewditu Memorial Hospital, Addis Ababa, Ethiopia* examines how local knowledge, beliefs, and perceptions related to halitosis are associated with oral health behaviors. The findings suggest that oral health literacy extends beyond information provision, encompassing how individuals understand, interpret, and apply health information within their social and experiential contexts. These studies highlight the importance of considering cultural perspectives and lived experiences when designing and implementing person-centered oral health education strategies.

## Educational and systemic determinants of oral health behavior

The collection also examines how educational environments and professional training influence oral health attitudes and clinical norms. In *Evaluating the role of dental education in shaping aesthetic preferences and clinical choices in Cambodia*, differences in aesthetic perceptions and reported clinical preferences between dental and non-dental students suggest that formal dental education can affect how individuals conceptualize dental appearance and related behaviors. Dental students demonstrated greater sensitivity to specific aspects of dental aesthetics, while non-dental students expressed higher overall satisfaction and stronger intentions toward aesthetic treatments, indicating variation in expectations and priorities that may be shaped by educational background. These findings support the view that educational and training contexts contribute to the development of professional attitudes and patient expectations, complementing efforts to enhance oral health literacy within broader community and clinical settings.

## Epidemiology and research trends

Beyond individual behavior and education, the collection extends to interdisciplinary and population-level perspectives. *Interdisciplinary research on periodontitis and depression: a bibliometric analysis of research trends, hotspots and future directions* maps the evolution and structure of scientific research at the intersection of periodontal disease and depression. By identifying publication trends, collaborative networks, and thematic clusters, the study highlights growing scholarly interest in the links between oral and mental health, without asserting causal mechanisms. This bibliometric perspective supports the value of considering oral health within broader, multidimensional research frameworks that integrate biological and psychosocial domains.

Meanwhile, the global analysis presented in *Global, regional, and national burden of orofacial clefts, 1990–2021* situates congenital oral conditions within a comprehensive epidemiological context. By documenting temporal trends and geographic variation in the burden of orofacial clefts, the study underscores substantial regional disparities in disease burden and health outcomes. These findings point to the need for context-sensitive prevention, care, and educational strategies, particularly in settings with limited resources, where structural and informational gaps may influence long-term health and quality of life.

Taken together, the nine contributions to this Research Topic highlight the interconnected roles of function, context, education, and systems in advancing oral health literacy and related outcomes. The studies emphasize the importance of moving beyond disease-based indicators to incorporate functional and quality-of-life–related outcomes, including psychosocial dimensions, as meaningful endpoints in both research and practice. They also demonstrate that oral health literacy is shaped by cultural, social, linguistic, and technological contexts, underscoring the need for literacy strategies that are locally grounded, community-engaged, and responsive to the lived realities of diverse populations.

At the same time, the findings point to the influence of educational and professional environments in shaping oral health norms, expectations, and communication practices, suggesting that literacy must be fostered not only among individuals and communities but also within training and clinical settings where future care practices are formed. Several contributions further support the integration of oral health within broader interdisciplinary and health system frameworks, recognizing that literacy-related outcomes are influenced by interactions between oral health, general health, and social determinants.

The collection draws attention to persistent global and local inequities in oral health outcomes, highlighting the need for equity-oriented policies that strengthen access to preventive care, culturally appropriate information, and supportive community resources. While technological and digital innovations emerge as valuable tools for expanding reach and engagement, the studies collectively caution that such approaches must be implemented with careful consideration of access, digital capacity, and cultural acceptability to avoid reinforcing existing disparities.

## Conclusion

The Research Topic *Enhancing Oral Health Literacy and Quality of Life: Strategies for a Healthier Future* advances understanding of oral health literacy as a multidimensional influence on both individual and population health. By bringing together evidence on oral function, community-engaged interventions, educational contexts, and global patterns of oral health, the collected studies highlight the potential of literacy-oriented approaches to support informed decision-making, strengthen care practices, and contribute to improved quality of life when implemented in context-sensitive and equitable ways.

Looking ahead, quality-of-life outcomes and functional measures should remain central considerations in oral health research, clinical practice, and policy development. Progress toward a healthier future will depend on sustained attention to literacy, system integration, and equity, alongside recognition that oral health is an essential component of overall well-being and social participation.
